# Insecticide Resistance Profiling of *Anopheles coluzzii* and *Anopheles gambiae* Populations in the Southern Senegal: Role of Target Sites and Metabolic Resistance Mechanisms

**DOI:** 10.3390/genes11121403

**Published:** 2020-11-25

**Authors:** Oumou. K. Gueye, Magellan Tchouakui, Abdoulaye K. Dia, Mouhamed B. Faye, Amblat A. Ahmed, Murielle J. Wondji, Daniel N. Nguiffo, Leon. M. J. Mugenzi, Frederic Tripet, Lassana Konaté, Abdoulaye Diabate, Ibrahima Dia, Oumar Gaye, Ousmane Faye, El Hadji A. Niang, Charles S. Wondji

**Affiliations:** 1 Laboratoire d’Écologie Vectorielle et Parasitaire, Faculté des Sciences et Techniques Université Cheikh Anta Diop, Dakar BP 5005, Senegal; milakd1@gmail.com (A.K.D.); elbachirfaye@gmail.com (M.B.F.); amblatali@hotmail.fr (A.A.A.); konatela@yahoo.fr (L.K.); jogomaye@yahoo.fr (O.F.); eaniang1@gmail.com (E.H.A.N.); 2 Centre for Research in Infectious Diseases (CRID), Yaounde BP 13591, Cameroon; mtchouakui@yahoo.fr (M.T.); murielle.wondji@lstmed.ac.uk (M.J.W.); daniel.ngiffo@crid-cam.net (D.N.N.); leon.mugenzi@crid-cam.net (L.M.J.M.); 3 Vector Biology Department, Liverpool School of Tropical Medicine, Pembroke Place, Liverpool L3 5QA, UK; 4 Centre for Applied Entomology and Parasitology, School of Life Sciences, Keele University, Newcastle-under-Lyme ST5 5BG, UK; f.tripet@keele.ac.uk; 5 Centre Muraz/Institut de Recherche en Sciences de la Santé, Bobo-Dioulasso BP 545, Burkina Faso; npiediab@gmail.com; 6 Pôle de Zoologie Médicale, Institut Pasteur de Dakar, 36 Avenue Pasteur, Dakar BP 220, Senegal; dia@pasteur.sn; 7 Service de Parasitologie-Mycologie, Faculté de Médecine, Pharmacie et d’Odontologie, Université Cheikh Anta Diop, Dakar BP 5005, Senegal; oumar.gaye@ucad.edu.sn

**Keywords:** malaria, *An. coluzzii*, *An. gambiae*, pyrethroid, *kdr*, N1575Y, metabolic resistance, Senegal

## Abstract

The emergence and spread of insecticide resistance among the main malaria vectors is threatening the effectiveness of vector control interventions in Senegal. The main drivers of this resistance in the *Anopheles gambiae* complex (e.g., *An. gambiae* and *Anopheles coluzzii*) remains poorly characterized in Senegal. Here we characterized the main target site and metabolic resistances mechanisms among the *An. gambiae* and *An. coluzzii* populations from their sympatric and allopatric or predominance area in Senegal. Larvae and pupae of *An. gambiae* s.l. were collected, reared to adulthood, and then used for insecticides susceptibility and synergist assays using the WHO (World Health Organisation) test kits for adult mosquitoes. The TaqMan method was used for the molecular characterization of the main target site insecticide resistance mechanisms (Vgsc-1014F, Vgsc-1014S, N1575Y and G119S). A RT-qPCR (Reverse Transcriptase-quantitative Polymerase Chaine Reaction) was performed to estimate the level of genes expression belonging to the CYP450 (Cytochrome P450) family. *Plasmodium* infection rate was investigated using TaqMan method. High levels of resistance to pyrethroids and DDT and full susceptibility to organophosphates and carbamates where observed in all three sites, excepted a probable resistance to bendiocarb in Kedougou. The L1014F, L1014S, and N1575Y mutations were found in both species. Pre-exposure to the PBO (Piperonyl butoxide) synergist induced a partial recovery of susceptibility to permethrin and full recovery to deltamethrin. Subsequent analysis of the level of genes expression, revealed that the *CYP6Z1* and *CYP6Z2* genes were over-expressed in wild-resistant mosquitoes compared to the reference susceptible strain (Kisumu), suggesting that both the metabolic resistance and target site mutation involving *kdr* mutations are likely implicated in this pyrethroid resistance. The presence of both target-site and metabolic resistance mechanisms in highly pyrethroid-resistant populations of *An. gambiae* s.l. from Senegal threatens the effectiveness and the sustainability of the pyrethroid-based tools and interventions currently deployed in the country. The *Kdr-west* mutation is widely widespread in *An. coluzzii* sympatric population. PBO or Duo nets and IRS (Indoor Residual Spraying) with organophosphates could be used as an alternative measure to sustain malaria control in the study area.

## 1. Introduction

The control of malaria vector in Africa relies mainly on the two core insecticide-based interventions: Long-Lasting Insecticide Nets (LLINs) and Indoor Residual Spraying (IRS) [1]. Four main classes of insecticide are available for use in the public health (pyrethroids, organochlorines, organophosphates and carbamates), with pyrethroids being the main class approved for the impregnation of nets [2]. Given the heavy reliance on pyrethroid-based strategies for malaria vector control, the spread across sub-Saharan Africa of the resistance to this class of insecticide threatens the sustainability of current and future vector control interventions. Thus, providing accurate and timely information about the evolution of the main insecticide resistance mechanisms is vital for the implementation of targeted and cost-effective control measures.

Two main types of mechanisms are involved in the resistance of *An. gambiae* to the principal classes of insecticide use in the public health sector: the target-site insensitivity [3,4] and the metabolic activity of detoxification enzyme families such as cytochrome P450s, glutathione S-transferases and esterases [5]. Target site insensitivity to DDT and pyrethroid in *An. gambiae* is associated to single-point mutation at the 1014 position in the voltage-gated sodium channel gene (*Vgsc*) known as knock-down resistance (*kdr*). The Vgsc-1014F and Vgsc-1014S mutations, respectively known as *kdr*-*west* and *kdr*-*east* [6,7], are among the most widespread target-site insecticide resistance mechanisms found among the natural population of *An. coluzzii* and *An. gambiae* [8] across the Western and Eastern Africa [4,9,10,11,12]. Previous studies in West Africa reported the absence of the *kdr* mutation in *An. coluzzii* even in the sympatric population [6,7,13]. However, subsequently, this mutation was found in both species with higher frequency of L1014F mutation in *An. coluzzii* [14] suggesting a introgression from *An. gambiae* to *An. coluzzii* [15]. The G119S-Ace-1 mutation is involved in bendiocarb resistance in *An. gambiae* s.l. in West Africa [16,17] and the N1575Y confer resistance to DDT and pyrethroid in west Africa [18,19].

In Senegal, four members of the *An. gambiae* complex were described so far, including the two incipient species *An. coluzzii* and *An. gambiae*, the two main malaria vectors across the continent. It have been reported in 2016 the presence and wide distribution of the Vgsc-1014F mutation among the wild populations of *An. gambiae* and *An. coluzzii* from a sympatric area in the south-eastern part of the country [20]. However, few data are available on the frequency and distribution of the Vgsc-1014F and Vgsc-1014S mutations in areas where one of the two species is predominant (allopatric area). The Moreover, few if any study has taken a holistic approach to characterize altogether the main target sites (Vgsc-1014F, Vgsc-1014S, N1575Y and G119S) as well as the putative metabolic mechanism. This infer the evolutionary processes underlying the emergence and spread of insecticide resistance among the natural populations of the two incipient species of the *An*. *gambiae* complex (e.g., *An. gambiae* and *An. coluzzii*) across their different range of distribution.

Here, we characterized the main target site and metabolic resistance mechanisms among the natural populations of *An. gambiae* s.s. and *An. coluzzii* in two ecogeographical regions of Senegal. The introgression of the *kdr-west* mutation from *An. gambiae* to *An. coluzzii* in the sympatric area was also assessed.

## 2. Materials and Methods

### 2.1. Study Areas

This study was conducted during the 2017 and 2018 raining seasons in three health districts located in two different eco-geographical zones of Senegal.

The districts of Tambacounda (13°46′14″ N; 13°40′02″ W) and Kedougou (12°33′28″ N; 12°10′27″ W) are both located in the southern region of the country and belong to the Sudanese eco-geographical zone. The health district of Fatick (14°21′29″ N; 16°35′08″ W is in the Sudan-Sahelian ecozone in the centre of Senegal. Tambacounda is located along the Gouloumbou River and was chosen as a sympatric area of the two incipient species. The main activity is agriculture of banana and rice involving high pesticide use. Kedougou is characterized by an important raining season with temporary breeding sites and was chosen as an allopatric area (or area of predominance) for *An. gambiae* whereas Fatick characterized by a low and irregular rainfall with a permanent river (Nema) was retained as an allopatric area (or area of predominance) for *An. coluzzii*. Subsistence crops are practiced in both areas with insecticide used.

### 2.2. Samples Collection

Larvae and pupae of *An. gambiae* s.l. were collected from breeding sites, during the two successive rainy seasons (August–October) in 2017 and (October–November) in 2018 and reared until emergence then exposed to insecticides. In addition, resting adult mosquitoes were collected indoor using the pyrethrum spray collection method, early on the morning (6:00 to 8:00 am), once every surveyed month during the all the study period (August–November).

### 2.3. Plasmodium spp. Infection Rate

Taqman method described by Bass [21] was used to screen samples for the presence of the *Plasmodium* spp. on the real-time PCR MX 3005 machine (Agilant, Santa Clara, CA, USA). The PlasF (5′-GCT TAG TTA CGA TTA ATA GGA GTA GCT TG-3′) and PlasR (5′-GAA AAT CTA AGA ATT TCA CCT CTG ACA-3′) primers set were used together with two probes labeled with the FAM fluorophore (Falcip+ 5′-TCT GAA TAC GAA TGT C-3′) to detect *Plasmodium falciparum*, and the HEX fluorophore (OVM+ 5′-CTG AAT ACA AAT GCC-3′) to detect *Plasmodium ovale*, *Plasmodium vivax* and *Pplasmodium malariae*. All positives samples were confirmed by nested PCR [22].

### 2.4. WHO Insecticide Susceptibility and Synergist Tests

Non-blood-fed females of *An. gambiae* s.l. aged of 3–5 days were exposed to DDT (4%), deltamethrin (0.05%), permethrin (0.75%), alphacypermethrin (0.1%), lambda-cyhalothrin (0.05%), bendiocarb (0.1%) and pirimiphos methyl (1%) using the standard WHO-susceptibility test procedures for adult mosquitoes at a temperature of 25 ± 2 °C and at 80 ± 10% relative humidity [23].

To investigate the putative role of detoxification enzyme in the pyrethroid resistance among highly resistant populations of *An. gambiae* s.l. from Kedougou, 3–5 days non-blood-fed females were tested against permethrin and deltamethrin as described above, after 1-h pre-exposure to 4% of Piperonal butoxide (PBO). For each insecticide molecules a batch of at least 50 specimens of 3–5 days non-blood-fed females were exposed to untreated papers as control.

Knock-downed specimens were recorded at 10, 15, 20, 30, 40, 50- and 60-min post exposure, and mortality was measured after a period of observation 24 h post-exposure.

### 2.5. Estimation of Resistance Intensity

To establish the intensity of pyrethroid resistance in Kedougou and Tambacounda, additional bioassays were conducted with 1×, 5× and 10× of the discriminating concentration of deltamethrin (0.05, 0.25, and 0.5%) and permethrin (0.75, 3.75, and 7.5%) as described by the standard protocol of WHO-susceptibility test procedures for adult mosquitoes [19].

### 2.6. Morphological and Molecular Identification of An. gambiae s.l. Species

All specimens collected indoor and those exposed to insecticides were identified using the Afrotropical Anopheline morphological keys of Gillies & de Meillon [24]. A sub-sample of indoors resting adult females, with together dead and alive specimens from insecticide susceptibility tests were randomly selected by area for subsequent analyses. 

The genomic DNA was extracted from single mosquito’s wings and legs using the Livak method [25], then the members of the *An. gambiae* complex were identified by the PCR [26,27]

### 2.7. Molecular Genotyping of the Vgsc-1014F, Vgsc-1014S, N1575Y and G119S Mutations

TaqMan assays were performed on the Agilent MX3005P qPCR (quantitative Polymerase Chain Reaction) system (Agilent, Santa Clara, CA, USA) to characterize the putative target site insecticide resistance mechanisms, including the Vgsc-1014F (West) and Vgsc-1014S (East) *Kdr* mutations [28], the N1575Y mutation [18] and the G119S Ace-1 mutation [29].

### 2.8. Analysis of the Polymorphism of the Voltage-Gated Sodium Channel

To assess the genetic diversity and detect putative mutations associated with the knockdown resistance (*kdr*), a fragment of 1014 of the voltage-gated sodium channel gene spanning the 1014 coding was analysed. This fragment which includes a portion of intron 19 and the entire exon 20 in the domain II of the segment 6 was amplified, purified, and sequenced in wild *An*. *gambiae* s.l. populations sampled in 2017 in Kedougou (12 *An. gambiae*, 6 *An. coluzzii,* and 4 hybrids), and Tambacounda (12 *An. gambiae*, 11 *An. coluzzii* and 4 hybrids). 

The genomic DNA was extracted from legs and wings as described by Livak [25] then amplified using the kdr-CL primers set (kdr-CL-F: 5′-AAATGTCTCGCCCAAATCAG-3′ and kdr-CL-R: 5′-GCA CCTGCAAAACAATGTCA-3′) as described by Pinto [30]. PCR products were purified using the exonuclease Ι (Exo Ι)/Shrimp Alkaline Phosphate (Exo-SAP) purification Kit (New England Biolabs, MA, USA) according to the manufacturer’s instructions, and sequenced using the ABI automated sequencer (Applied Biosystems, Foster City, CA, USA).

The amplified sequences were corrected using BioEdit v.7.2.1 [31] then aligned using ClustalW [32]. Phylogenetic analysis and haplotype reconstruction were done using the DnaSP v.5.10 [33]. Sequences were compared with reference sequences retrieved from Genbank (http://blast.ncbi.nlm.nih.gov/Blast.cgi) and the maximum likelihood phylogenetic tree was constructed using MEGA v.7.0 [34]. 

### 2.9. Metabolic Resistance Genes Expression

The expression level of the CYP450 genes family (*CYP6M2*, *CYP6P3*, *CYP4G16*, *CYP4G16*, *CYP9K1*, *CYP6Z1*, and *CYP6Z2*), and *GSTe2* was assessed from three biological replicates of surviving *An. gambiae* after exposure to Permethrin (Kedougou and Tambacounda) and Deltamethrin (Kedougou). RNA (Rubonucleic acid) was extracted and purified using the picopure RNA isolation Kit (Life Technologies, Camarillo, CA, USA) according to the manufacturer’s instructions. cDNA (complementary Deoxyribonucleic acid) was synthesized from the purified RNA by quantitative RT-PCR using the SuperScript III (Invitrogen, Waltham, MA, USA) and the oligo-dT20 and RNAse H (New England Biolabs, Ipswich, MA, USA) kit in a total reactional volume of 20 μL including of 19 μL PCR mix (10 μL of SyBr Green, 7.8 μL of dH_2_O, 0.6 μL of forward and reverse primers at the concentration of 10 mM for each gene of interest), and 1 μL of cDNA (or dH_2_O water for controls). Amplification was performed with an initial step of denaturation at 95 °C for 3 min followed by 40 cycles of 10 s at 95 °C, 10 s at 60 °C, then one cycle of 1 min at 95 °C, 30 s at 55 °C and 30 s at 95 °C. The cDNA extract from the *An. gambiae* Kisumu susceptible strain was used as a susceptible biological control.

### 2.10. Data Analysis

The 24 h post-exposure mortality for bioassay was estimated for each insecticide tested by dividing the number of dead mosquitoes per replicate by the total number of mosquitoes exposed. Odds ratios, Chi-square and Fisher’s exact tests were used for statistical comparisons. The relative expression for each metabolic gene was calculated according to the 2^−ΔΔCT^ method [35] and the statistical significance between gene expression estimates was performed using unpaired Student *t* test. The 5% significance level was considered for all the statistical tests. All analyses were conducted using GraphPad Prism version 7.00 and R version 3.5.2 software version.

## 3. Results

### 3.1. Species Composition

#### 3.1.1. Indoor Collection

A total of 1474 specimens were collected, 657 and 817 in 2017 and 2018, respectively. In the *An. gambiae* complex, *An. arabiensis* was predominant in Fatick (81.68 vs. 65.17%) and was present in Kedougou (16.36 vs. 7.46%) and Tambacounda (28.94 vs. 15.64%). *An. melas* was found only in Fatick (Table S1). Compared to *An. gambiae*, *An. coluzzii* was most abundant in Fatick (67.85% vs. 88.52%) but less abundant in Kedougou (93.99% vs. 90.32%) in 2017 and 2018 respectively (Table S1). In Tambacounda, considered as the sympatric area of *An. coluzzii* and *An. gambiae*, the latter was found predominant. Hybrids *An. coluzzii*/*An. gambiae* were found in all areas in 2018 with frequencies ranging from 1.64 to 3.57% except in Kedougou (Table S1). 

#### 3.1.2. Larval Collection

A total of 1091 specimens were identified, *An. arabiensis* was the predominant species in Fatick and Tambacounda (Table S2). When considering the two incipient species *An. coluzzii* and *An. gambiae*, the latter was predominant in Kedougou (86.64%). In Tambacounda *An. coluzzii* and *An. gambiae* were found almost at the same proportion (50% and 47.03% respectively). In Fatick, *An. gambiae* was found predominant (84.85%) (Table S2).

### 3.2. Plasmodium spp. Infection Rate 

DNA was extracted from 314 mosquitoes (head-thorax) collected in 2017 and 2018 and analyzed using TaqMan assay for *Plasmodium* infection. In Kedougou, 3.16% (3/95) mosquitoes were found infected with *Plasmodium ovale*, *vivax* or *malariae* and 1.05% (1/95) infected with *Plasmodium falciparum*. All the mosquitoes infected were *An. gambiae.* In Tambacounda, 2.53% (4/158) of mosquitoes were found infected with *P. falciparum* and 0.63% (1/158) were co-infected with *P. falciparum* and *P. ovale* or *vivax* or *malariae* (OVM+). Among these infected mosquitoes, 1.27% (2/158) were *An. gambiae,* 0.63% (1/158) were *An. coluzzii* and 0.63% (1/158) was hybrid *An. coluzzii*/*An. gambiae.* The co-infected mosquitoes were identified as *An. coluzzii*. In Fatick, no mosquito was found infected. The nested PCR confirmed all *Plasmodium falciparum* positive mosquitoes, but failed to confirm the OVM+ from TaqMan probably because of the low sensitivity of this method [21].

### 3.3. Insecticide Resistance Profile

**Figure 1:**
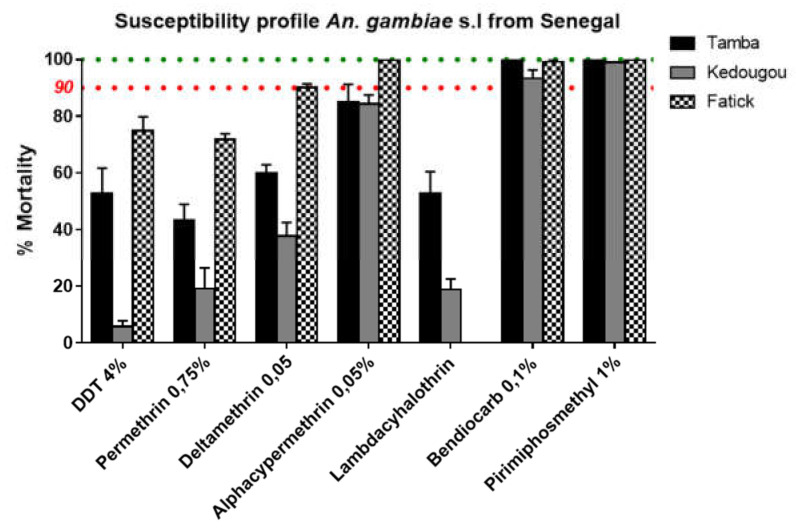
Susceptibility profile of *Anopheles gambiae* s.l. to insecticides. Recorded mortalities following 60-min exposure of *Anopheles gambiae* s.l. from Fatick, Tambacounda and Kedougou to different insecticides. Data are shown as mean ± standard error of the mean (SEM).

A total of 2141 mosquitoes from the 2017 collection were tested for the conventional WHO bioassay including 655 from Fatick, 262 from Kedougou, and 724 from Tambacounda. Mosquitoes tested were fully susceptible to bendiocarb and pirimiphos methyl. However, in all Kedougou probable resistance to bendiocarb was noted with 93.3 ± 3% (SEM) mortality (Figure 1). High level of resistance to DDT (5.8 ± 2%; 52.9 ± 8%), permethrin (19.1 ± 7.4%; 43.3 ± 5.6%), deltamethrin (37.7 ± 4.8%; 60 ± 2.8%), lambda-cyhalothrin (18.9 ± 3.6%; 52.9 ± 7.5%) and alphacypermethrin (84.3 ± 3.2%; 85.1 ± 6.2%) was recorded in Kedougou and Tambacounda respectively (Figure 1). However, in Fatick, full susceptibility to alphacypermethrin and probable resistance to deltamethrin (90.2 ± 1.2%) were observed whereas moderate resistance was noted for DDT (74.8 ± 5%) and permethrin (71.8 ± 2%) (Figure 1).

### 3.4. Estimation of Resistance Intensity 

**Figure 2:**
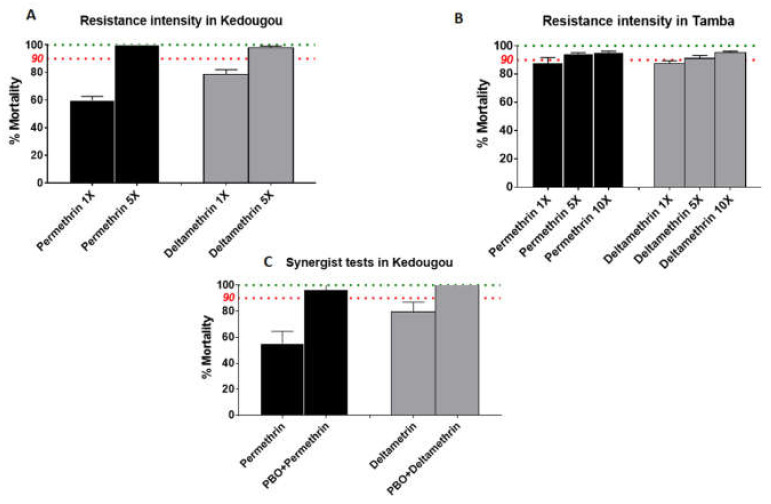
Results of resistance intensity and synergist tests. Resistance intensity in Tambacounda (**A**) and Kedougou (**B**); activities of PBO combined to permethrin, and deltamethrin on *An*. *gambiae* s.l. from Kedougou (**C**). Data are shown as mean ± standard error of the mean.

To assess the strength of the phenotype resistance to permethrin and deltamethrin, the resistant population collected in 2018 from Kedougou and Tambacounda were exposed to 5× and 10× of discriminating concentration of permethrin and deltamethrin. Results showed a low intensity of resistance to permethrin (5×: 100%) and deltamethrin (5×: 98 ± 1.1%) in Kedougou (Figure 2A) whereas in Tambacounda a higher intensity of resistance to permethrin (5×: 94.3 ± 0.9%; 10×: 95.3 ± 0.9%) and deltamethrin (5×: 91.2 ± 2%; 10×: 95.3 ± 0.9%) were found (Figure 2B).

### 3.5. Synergist Bioassay with PBO

To assess the implication of the cytochrome P450s in the resistance observed to permethrin and deltamethrin, mosquitoes collected in 2018 from Kedougou were pre-exposed to PBO then to permethrin or deltamethrin. Compared to the result of the permethrin alone (mortality: 55.42 ± 9.19%) a nearly full recovery of the susceptibility was observed after exposure to permethrin + PBO (mortality: 96.47 ± 9.19%). For deltamethrin, a total recovery of the susceptibility was observed after pre-exposure to the PBO (mortality: 100%) compared to the result of deltamethrin alone (mortality: 79.74 ± 7.16%) (Figure 2C).

### 3.6. Distribution of Resistance Markers in the Adult Mosquitoes Collected 

**Figure 3:**
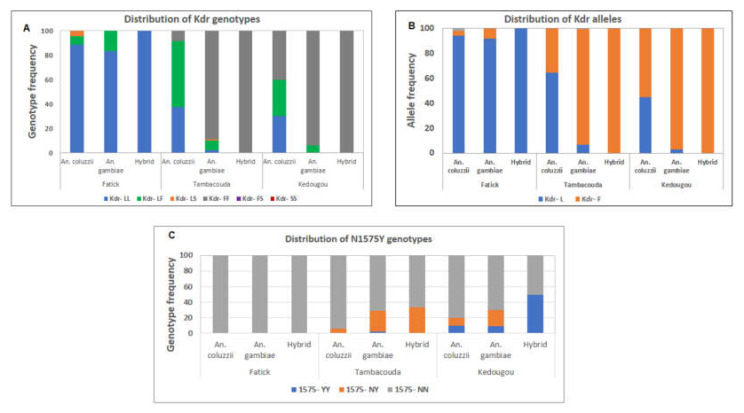
Genotyping of resistance markers in *An. coluzzii*, *An. gambiae* s.s. and their hybrids. Distribution of Kdr(s) genotypes (**A**) and alleles (**B**) and N1575Y genotypes (**C**) in the different species. Kdr − LL = Kdrw susceptible homozygous; Kdr − LF = Kdrw heterozygous; Kdr − FF = Kdrw resistant homozygous; Kdr − LS = Kdre resistant heterozygous; Kdr − SS = Kdre resistant heterozygous; Kdr − FS = Kdrw and Kdre resistant; 1575 − YY = Resistant homozygous; 1575−NY = Resistant heterozygous; 1575 − NN = Susceptible homozygous.

In all the three sites, the L1014F mutation was found in both species. In Kedougou, the predominance area of *An. gambiae*, all *An. gambiae* mosquitoes (64/64) harboured the mutation whereas only 70% (7/10) of the *An. coluzzii* harboured it *(*Figure 3A). The frequency of the L1014F resistant allele was higher in *An. gambiae* (96.88%) compared to *An. coluzzii* (55%) (*χ*^2^ = 18.9; *p* < 0.0001) (Figure 3B). No difference was found between the frequency of the L1014F resistant allele in *An. gambiae* from this site compared to Tambacounda (*χ*^2^ = 0.79, *df* = 1, *p* = 0.37).

The N1575Y mutation was also found in both species with a frequency of 29.69% (19/64) in *An. gambiae* and 20% (2/10) in *An. coluzzii* (Figure 3C), The L1014S mutation was absent in this area (Figure 3A).

As observed in Kedougou, the frequency L1014F mutation was higher in *An. gambiae* 96.63% (86/89) from Tambacounda compared to *An. coluzzii* 62.5% (30/48) (*χ*^2^ = 28.7; *p* < 0.0001) (Figure 3A,B). Moreover, a significant difference was found also when comparing the distribution of this mutation in *An. coluzzii* from Fatick compared to Tambacounda (*χ*^2^ = 28.57, *df* = 1, *p* < 0.001). 

The N1575Y mutation was found at 29.21% (26/89) in *An. gambiae* and at 6.25% (3/48) in *An. coluzzii.* Only the heterozygote (N1575Y) was detected in *An. coluzzii* (Figure 3C). The L1014S mutation was found in only *An. gambiae* at the heterozygote form as well (Figure 3A). 

In Fatick, the predominance area of *An. coluzzii*, the L1014F mutation was at 6.82% (3/44) in this species and at 16.67% (1/6) in *An. gambiae (*Figure 3A). The N1575Y mutation was not found in this area (Figure 3C) and only two *An. coluzzii* were found carrying the L1014S mutation (Figure 3A).

All the hybrids genotyped in Kedougou (*n* = 2) and Tambacounda (*n* = 6) harboured the L1014F mutation (Figure 3A). In Kedougou, 50% of them carried the N1575Y mutation whereas only 33.33% in Tambacounda carried this mutation (Figure 3C). 

### 3.7. Correlation between the 1014F Mutation and Resistance to Pyrethroid

To assess the implication of *kdr-w* mutation in the pyrethroid resistance observed in *An. gambiae*, an allelic and genotypic association analysis was performed on 110 individuals, including 71 alive and 39 dead after exposition to pyrethroids in Kedougou. Pearson correlation test showed no significant association between pyrethroid resistance and the presence of L1014F resistant allele (*Odds Ratio* = 3.7 (95% CI: 0.7–18.2, *p* = 0.08)). This was confirmed when comparing the likelihood of surviving of females with RR genotypes to survive compared to RS (*Odds Ratio* 1.3 (95% CI: 0.3–5.1, *p* = 0.2)), and SS (*Odds Ratio* 5.3 (95% CI: 0.6–46.5, *p* = 0.1)). The same pattern was observed between RS and SS (*Odds Ratio* 4.0 (95% CI: 0.3–49.6, *p* = 0.3)) (Table 1). The association between pyrethroid resistance and L1014F mutation was not assessed in the other locality due to the low number of dead *An. coluzzii* and *An. gambiae*. 

### 3.8. Genetic Diversity in the kdr Locus of the Voltage-Gated Sodium Channel

**Figure 4:**
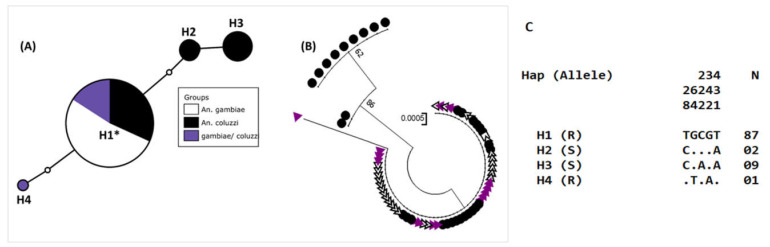
Genetic diversity parameters of Vgsc in *An. coluzzii*, *An. gambiae and* their hybrids from Senegal in relation to the species. (**A**) Haplotype network in relation to the species composition; (**B**) phylogenetic trees (using a maximum likelihood method) and the nucleotide diversity of the L1014F mutation in Senegal (**C**).

A total of 484-bp fragments of the *VGSC* spanning the 1014 codon were successfully sequenced in 24 *An. gambiae,* 17 *An. coluzzii and 8 An. coluzzii/An. gambiae* from Kedougou and Tambacounda. The genetic diversity parameters of this fragment is provided in the Table S3. Overall, five polymorphic sites defining 4 haplotypes were detected with a haplotypic diversity of 0.219. The overall nucleotide diversity was 0.001. At the species level, low haplotypic and genetic diversity were found in *An. coluzzii* and the hybrids. Analysis of the haplotype Network showed that the major and ancestral haplotype H1 (87/99) was shared between *An. coluzzii*, *An. gambiae* and their hybrids and was specific to the L1014F resistant allele. The two following haplotypes H3 (9/99) and H2 (2/99) were specific to L1014F susceptible allele and carried by *An. coluzzii* only. The lowest H4 (1/99) belonged to the L1014F resistant allele and was specific to the hybrid (Figure 4A,C).

The analysis of the maximum likelihood phylogenetic tree between mosquitoes from different localities showed two main clades: the major with the two species and their hybrids and the second made up only by *An. coluzzii* (Figure 4B).

### 3.9. Implication of the G119S Mutation in the Observed Bendiocarb Resistance

To assess the implication of the G119S mutation in the moderate resistance to bendiocarb observed in Kedougou, 7 mosquitoes alive to bendiocarb and 16 dead after exposition were genotyped. All dead samples were homozygous susceptible (G/G119) and among the 7 alive the only one which was amplified was homozygous resistant (119S/S). This low sample size did not allow to draw a conclusion on the role of this mutation in the resistance to bendiocarb in this area. However, genotyping of the G119S-Ace1 marker in 62 field-collected mosquitoes revealed the presence of resistant allele with the frequency of 22.58%. This frequency of the resistant allele suggest that this mutation could be involved in this resistance to carbamates.

### 3.10. Expression Profiling of Metabolic Genes

**Figure 5:**
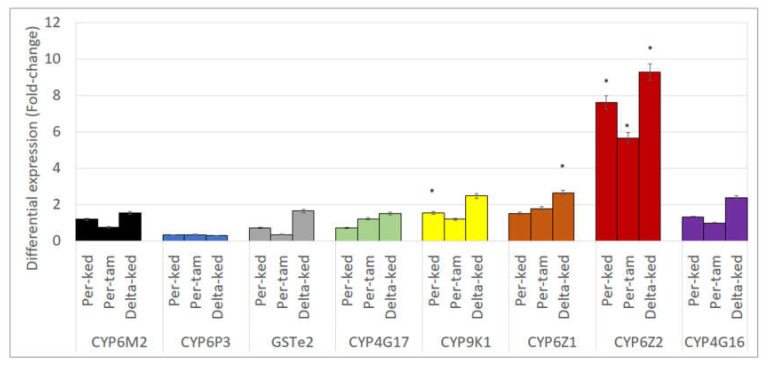
Differential expression by quantitative reverse-transcription polymerase chain reaction of the major insecticide resistance genes in *An. gambiae* in Senegal compared with the susceptible Kisumu. Error bars represent standard error of the mean. *—statistically significant *p* ≤ 0.05.

The expression level of *CYP6M2*, *CYP6P3*, *CYP4G16*, *CYP4G17*, *CYP9K1*, *CYP6Z1*, *CYP6Z2* and *GSTe2* was evaluated in *An. gambiae* from Kedougou and Tambacounda using the susceptible *An. gambiae* laboratory strain (Kisumu) as a control. The results showed no difference in the expression level of *CYP6M2*, *CYP6P3*, *CYP4G16*, *CYP4G17*, and *GSTe2* between Kisumu and the field-collected *An. gambiae.* Only *CYP6Z2* was found highly overexpressed, in Deltamethrin (Fold-change (*FC*) = 9.26 ± 4.99) (*t* = 2.4; *df* = 4; *p* = 0.03) and Permethrin (*FC* = 7.62 ± 5.26) (*t* = 3.2; *df* = 4; *p* = 0.01) resistant mosquitoes from Kedougou compared to Kisumu. This gene tended also to be overexpressed (*FC* = 5.66 ± 0.14) in Permethrin resistant mosquitoes from Tambacounda compared to Kisumu (*t* = 1.9; *df* = 4; *p* = 0.05) like the *CYP9K1* gene in Permethrin resistant mosquitoes from Kedougou (*t* = 2.09; *df* = 4; *p* = 0.05). Furthermore, *CYP6Z1* was significantly overexpressed in Deltamethrin resistant mosquitoes from Kedougou (*FC*: 2.64 ± 0.29) (*t* = 2.7; *df* = 4; *p* = 0.02) (Figure 5).

## 4. Discussion

In this study the main aim was to determine the insecticide resistance profile and the distribution of *kdr* mutation in *An. coluzzii* and *An. gambiae* population from their sympatric and allopatric or predominance area in Senegal in the 2017 and 2018 raining season.

*An. gambiae was* predominant in rural and most humid areas (Kedougou and Tambacounda), while *An. coluzzii* was the most abundant in the arid area (Fatick). *An. coluzzii* and *An. gambiae* differ in their ecological preference both at the larval or adult stages [36] thus explaining their spatial and temporal distribution [37,38]. *An. gambiae* larvae are found in rain-dependent surface water bodies/puddles while those of *An. coluzzii* are more adapted to more permanent anthropogenic breeding sites such as irrigated rice fields [6,11,39,40,41]. Furthermore, *An. coluzzii* larvae displayed a greater tolerance to aridity and even organic pollution [42].

The low *Plasmodium* infection rate found in *An. coluzzii* and *An. gambiae* from Fatick and Tambacounda and Kedougou corroborate with other findings [43,44]. It could be due by the use of LLINs which reduce the human vector contact or cause a behavioural change of the vector. This was demonstrated in *An. gambiae* which was highly anthropophilic before the widespread use of nets showed a trophic deviation towards cattle [45].

Globally, the results of bioassays showed that the populations tested were resistant to DDT and Pyrethroids and susceptible to organophosphate and carbamate except in Kedougou where bendiocarb resistance was suspected. Previous studies in Senegal showed the same status of resistance in Tambacounda, Kedougou and other location [20,46,47]. However, in Fatick, as our findings, it was reported a susceptibility to bendiocarb and suspected resistance to deltamethrin but, they found a suspected resistance to pirimiphos methyl with what we have found a susceptibility [48]. In contrast to our findings, recently it was found a high resistance to bendiocarb and Pirimiphos methyl in an urban area in Senegal due to the large use of this molecule in the crops protection [9].

The resistance to DDT and pyrethroids is common in most African countries [6,40,47,49]. DDT resistance has often been linked to its historical use for vector-borne diseases and crop pest control. Despite the fact that this insecticide have been abandoned, DDT could persist in the environment due to its widespread use in public health and agriculture in the past decades [40,50]. Furthermore, the cross-resistance between pyrethroids and DDT through *kdr* could further explain the high DDT resistance. Resistance to pyrethroids could be due to the fact that they are the main molecules recommended for bed nets impregnation which is largely distributed across several African countries [2] as noted in Senegal [51,52,53,54]. In Kedougou food crops were practiced during the raining season which could involve the use of commercial pesticides comprising carbamate and pyrethroids. This could explain the suspected resistance to bendiocarb in this area.

The resistance level varied between the three sites. It was low in Fatick compared to Tambacounda and Kedougou. The resistance assay showed a high intensity of resistance in Tambacounda and moderate intensity in Kedougou. This could be explained by the fact that in addition to the LLNs use in Tambacounda, this site is an area with intense agriculture activity (banana, rice) with recurrent use of pesticides on crops throughout the year compared to Kedougou where subsistence crops are practicing only during the raining season. This finding corroborates with previous studies showing a significant correlation between agriculture intensity and phenotypic resistance in Tanzania [55]. Previous studies showed that in west Africa, pyrethroid resistance is high and predominant in *An. gambiae* compared to *An. arabiensis* [20,56] this could explain the decrease of pyrethroid resistance in 2018 compared to 2017. In this latter, bioassay was conducted in September–October when *An. gambiae* was predominant and in 2018 in October–November where *An. arabiensis* proportion became important.

The predominance of the L1014F mutation has been highlighted in *An*. *gambiae* from west Africa [6,12,57]. Previous studies have reported the presence of this mutation in *An. gambiae* only and not in *An. coluzzii* even in the sympatric areas [57,58]. This situation was found in 2009 in Tambacounda (Senegal) where no *An. coluzzii* carried the mutation [13]. However, subsequent study in this same area in 2016 by the same authors [20] reported the presence of the L1014F mutation in both species. In the present study, the findings corroborate with those of Niang [20] with the presence of the 1014F mutation in both species in the same area, but the frequency of the mutation was higher in our study. This support the results in Benin [10] and Mali [59] with a high frequency of the mutation in both species. 

In Kedougou the *An. gambiae* predominant area, the L1014F mutation was higher and tended to fixation in this population. However, in Fatick the predominance area of *An. coluzzii* 89% of this latter were susceptible. A very low frequency of the mutation in a predominant area of *An. coluzzii* was also found in previous studies in west Africa [6,60]. The presence of the L1014F mutation in *An. coluzzii* has been attributed to introgression from *An. gambiae* [15,61] and the results found here in the sympatric can support this hypothesis.

The L1014S mutation was detected only in *An. arabiensis* and *An. coluzzii* [9,48] in Senegal and only in *An. arabiensis* in Benin [10]. However, in this study, in addition to *An. coluzzii* (two) in Fatick we have also found one *An. gambiae* carrying the mutation but all at the heterozygote state. Similar result was also found in central Africa (in Equatorial Guinea [62] and in Cameroun [63,64]) 

The presence of the N1575Y mutation has been reported in West Africa [18,19,65]. This study was the first showing the presence of this mutation in Senegal It was found to be present in both *An. coluzzii* and *An. gambiae* in Tambacounda the sympatric area and Kedougou the predominance zone of *An. gambiae* where the frequency of the L1014F mutation was high. However, it was absent in Fatick, the predominance area of *An. coluzzii* where the frequency of the L1014F mutation was very low. This corroborates the results of Jones and collaborators reporting no N1575Y mutation in the samples from areas of low frequency of the L1014F mutation. In this study, the N1575Y mutation in both species were found exclusively in mosquitoes harbouring the L1014F mutation as found in Burkina Faso [19]. This finding supports the hypothesis that N1575Y mutation was linked to the L1014F mutation suggesting that the N1575Y mutation compensates for deleterious fitness effects of L1014F and/or confers additional resistance to insecticides [18].

The G119S-Ace-1 mutation is found to be involved in bendiocarb resistance in the *An. gambiae* s.l. in West Africa [16,17]. However, in this study the mutation is well present in the adult population, but further studies are needed to confirm its implication in the bendiocarb resistance in this area. 

As found in previous studies [66,67], the absence of correlation between the *kdrw* mutation and resistance to pyrethroid in the *An. gambiae* population from Kedougou is probably due to the fact that this resistance allele is already fixed in this location masking it’s role. Experiments performed here also suggest that metabolic resistance is playing an important role in this resistance. This was most evident based on the results of synergist bioassay with PBO showing a nearly or full recovery of the susceptibility to permethrin and deltamethrin respectively. Overexpression of P450 enzymes has been demonstrated to play a major role in pyrethroid resistance in insects [63] including in other malaria vectors such as *An. funestus* in Senegal [68]. Likewise, high GSTs activity was reported to be associated with insect resistance to DDT and pyrethroids [59,69]. The following candidate genes used in this study (*CYP6M2*, *CYP6P3* [70,71,72], *CYP6Z2* [73], *CYP4G16*, *CYP4G17* [19,74,75] and *CYP9K1*) have been reported to be involved in pyrethroid resistance in *An. gambiae* in Africa [76]. 

In this study only *CYP6Z1* and *CYP6Z2* have been differentially expressed between field-resistant mosquitoes and the susceptible strain suggesting a potential implication of these two genes in the pyrethroid resistance observed.

## 5. Conclusions

This findings of high pyrethroid and DDT resistance in *An. gambiae* and *An. coluzzii* from Senegal is a major obstacle to malaria control using pyrethroid or DDT-based tools. PBO or Duo nets and IRS with organophosphates could be used as an alternative measure to sustain malaria control in the study area as metabolic resistance was found implicated. Full susceptibility was noticed with organophosphate and carbamates. Our findings showed that the L1014F mutation is widespread in the sympatric *An. coluzzii* population and that the L1014S is present at very low frequency in both species. This study reveals for the first time the presence of the N1575Y mutation in *An. coluzzii* and *An. gambiae* in Senegal. Further studies are needed to better understand the evolution of this mutation and its implication to the resistance.

## Figures and Tables

**Table 1:** Association between L1014F-kdrw mutation and resistance to pyrethroids in *Anopheles gambiae* from Kedougou.

Combination of Genotypes at the L1014F-*kdr* Locus	*An. gambiae*	
	*Odds Ratio*	*p*-Value
**RR vs. RS**	1.3	0.2
	(0.3–5.1)	
**RR vs. SS**	5.3	0.1
	(0.6–46.5)	
**RS vs. SS**	4.0	0.3
	(0.3–49.6)	
**R vs. S**	3.7	0.08
	(0.7–18.2)	

## Data Availability

The datasets generated and/or analysed during the current study are available from the corresponding author upon request.

## Supplementary Materials

The following are available online at https://www.mdpi.com/2073-4425/11/12/1403/s1, Table S1. Species composition of the *An. gambiae s.l* collected indoor. Table S2. Species composition of adult from Larvae collection. Table S3. Genetic diversity parameter of voltage gate sodium channel gene.
