# Supplementary material for: Insecticide Resistance Profiling of Anopheles coluzzii and Anopheles gambiae Populations in the Southern Senegal: Role of Target Sites and Metabolic Resistance Mechanisms
Source: Genes (Basel). 2020 Nov 25;11(12):1403. doi: 10.3390/genes11121403 (PMC7760107; doi:10.3390/genes11121403)
Supplement: Supplementary file 1 [file genes-11-01403-s001.pdf]

Table S1. Species composition of the *An. gambiae s.l* collected indoor.

		**Proportion of the differents species of *An. gambiae complex***				**Proportion of the incipient species of *An. gambiae s.s.***			
**Year**	**Locality**	***An. arabiensis***	***An. melas***	***An. gambiae s.s.***	**N**	***An. coluzzii***	***An. gambiae***	***An. coluzzii/An. gambiae***	**N**
**2017**	Kedougou	36 (16.36%)	-	184 (83.64%)	**220**	9 (4.92%)	172 (93.99%)	3 (1.64%)	**184**
	Tambacounda	68 (28.94%)	-	167 (71.06%)	**235**	14 (8.28%)	148 (87.57%)	5 (2.96%)	**167**
	Fatick	165 (81.68%)	9 (4.46%)	28 (13.86%)	**202**	19 (67.85%)	8 (28.57%%)	1 (3.57%)	**28**
**2018**	Kedougou	10 (7.46%)	-	124 (92.54%)	**134**	12 (9.68%)	112 (90.32%)	-	**124**
	Tambacounda	79 (15.64%)	-	426 (84.36%)	**505**	99 (23.23%)	313 (73.47%)	14 (3.29%)	**426**
	Fatick	116 (65.17%)	1 (0.56%)	61 (34.27%)	**178**	54 (88.52%)	6 (9.84%)	1 (1.64%)	**61**

Abbreviations: *N total number of specimens collected; An. coluzzii/An. gambiae hybrid obtained after molecular identification by PCR.*

Table S2*:* Species composition of adult from Larvae collection.

	Proportion of the differents species of *An. gambiae complex*			Proportion of the incipient species of *An. gambiae*			
**Locality**	***An. arabiensis***	***An. gambiae***	**N**	***An. coluzzii***	***An. gambiae***	**An. coluzzii/An. gambiae**	**N**
**Kedougou**	38 (8.05%)	434 (91.95%)	**472**	53 (12.21%)	376 (86.64%)	5 (1.15%)	**434**
**Tambacounda**	185 (47.80%)	202 (52.20%)	**387**	101 (50%)	95 (47.03%)	6 (2.97%)	**202**
**Fatick**	199 (85.78%)	33 (14.22%)	**232**	5 (15.15%)	28 (84.85%)	-	**33**

Abbreviations: *N total number of specimens collected; An. coluzzii/An. gambiae hybrid obtained after molecular identification by PCR.*

Table S3. Genetic diversity parameter of voltage gate sodium channel gene.

	2N	S	h	Hd	π	D	D*
Kedougou *An. coluzzii*	12	3	2	0.409	0.002	0.77	1.105
Kedougou *An. gambiae*	24	0	1	0	0.000	/	/
Kedougou Hybride	8	1	2	0.25	0.001	-1.31^{ns}	-1.41^{ns}
Tamba *An. coluzzii*	22	3	3	0,498	0.002	1.427^{ns}	0.991^{ns}
Tamba *An. gambiae*	24	0	1	0	0.000	/	/
Tamba Hybride	8	0	1	0.000	0.000	/	/
Total	98	5	4	0.219	0.001	-0.804^{ns}	-1.088^{ns}

Abbreviations: 2*N*, number of sequences; S, number of polymorphic sites; h, haplotype; Hd, haplotype diversity; π, nucleotide diversity; D, Tajima's statistic; D*, Fu and Li's statistic; ns, not significant
